# Results of open thoracoabdominal aortic replacement in patients unsuitable for or after endovascular repair with remaining disease components

**DOI:** 10.1093/icvts/ivac076

**Published:** 2022-04-19

**Authors:** Stoyan Kondov, Leon Frankenberger, Matthias Siepe, Cornelius Keyl, Klaus Staier, Frank Humburger, Bartosz Rylski, Maximilian Kreibich, Tim Berger, Friedhelm Beyersdorf, Martin Czerny

**Affiliations:** 1 Department of Cardiovascular Surgery, Faculty of Medicine, University Heart Centre, University Hospital Freiburg, Albert-Ludwigs-University of Freiburg, Freiburg, Germany; 2 Faculty of Medicine, Albert Ludwigs University Freiburg, Freiburg, Germany; 3 Department of Anesthesiology & Critical Care Medicine, Medical Center - Faculty of Medicine, University of Freiburg, Freiburg, Germany

**Keywords:** thoracoabdominal replacement, open surgery, endovascular repair

## Abstract

**OBJECTIVES:**

Our goal was to evaluate outcomes in all-comer patients undergoing open thoracoabdominal aortic replacement either unsuitable for or after failed endovascular aortic repair.

**METHODS:**

Within a 4-year period, we analysed a consecutive series of 80 patients undergoing elective, urgent and emergency thoracoabdominal aortic replacement. Preoperative data, intraoperative data and outcomes were evaluated. Specific attention was given to technical refinements needed in patients after previous endovascular aortic repair.

**RESULTS:**

Eighty patients underwent thoracoabdominal aortic replacement: 11.3% (n = 9) had connective tissue disorders. Twenty-six patients (32.5%) had previous endovascular aortic repair and 54 (67.5%) did not have previous endovascular repair. The mean age was 64.2 ± 12 years, and 70% (n = 56) were male. The mean EuroSCORE was 7.9 ± 2.6. Urgent or emergency operations were done in 22.5% (n = 18). Overall mortality was 20% (n = 16); symptomatic spinal cord injury occurred in 5% (n = 4). We did not observe differences in survival according to the presence or absence of previous endovascular aortic repair (*P* = 0.524). Multivariate regression analysis revealed the amount of packed red blood cell units (*P* = 0.009, confidence interval 1.028–1.215, odds ratio = 1.117) as a predictor of in-hospital death. Follow-up was 100% (37.9 ± 15.8 months); freedom from aortic-related reintervention was 96.3%.

**CONCLUSIONS:**

Despite an early attrition rate, survival after open thoracoabdominal aortic replacement is excellent, and freedom from aortic-related reintervention is high. Open surgery continues to remain an essential component in the treatment armamentarium of acute and chronic thoracoabdominal aortic pathology.

**Figure:**
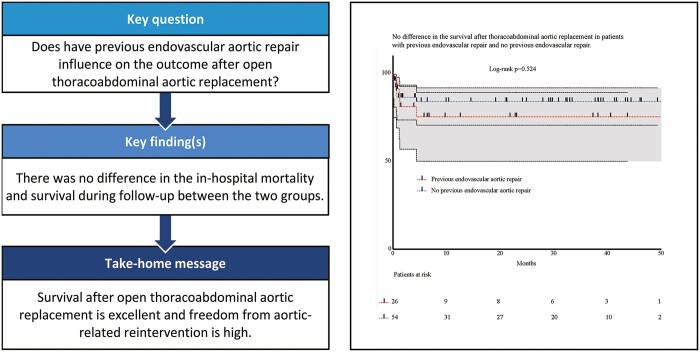

## INTRODUCTION

The number of patients with thoracoabdominal aortic pathologies warranting treatment is increasing due to the increasing number of patients with remaining dissection after previous type A repair [1–3]. Whereas open surgery has been the only treatment option for many years, endovascular aortic repair has emerged as an excellent alternative [4–6]. Although in-hospital mortality is lower, the need for aortic-related interventions is higher, and, finally, conversion to open surgery is needed in some cases [7]. The numbers of these types of cases are expected to rise in the years to come due to the substantial increase in the number of endovascular procedures being performed [8–10]. As the volume of endovascular procedures volume rises, the age and comorbidities of the patients increase as well. Open TAAA (thoracoabdominal aortic aneurysm) repair remains a major surgical procedure. In the modern endovascular era, it remains an option for young patients, patients with underlying connective tissue disorders and those who are not suitable for endovascular solutions [11]. Furthermore, the expertise in the different centres plays a major role in deciding which treatment strategy to choose and finally in the clinical outcome [4, 12].

The goal of this study was to evaluate outcomes in patients undergoing open thoracoabdominal aortic replacement who were either unsuitable for or who had a failed endovascular aortic repair.

## METHODS

### Ethics Statement

The ethical committee of the University Hospital Freiburg approved the study (558/19). The inormed consent was waived due to the retrospective nature of the study.

### Patients

Within a 4-year period (January 2015– December 2019), we analysed a consecutive series of 80 all comers undergoing elective, urgent and emergency thoracoabdominal aortic replacement. No patients were refused for anatomical reasons or for reasons regarding previous endovascular repair. Preoperative data, intraoperative data, outcome and survival and freedom from aortic-related interventions were evaluated. Specific attention was given to technical refinements needed in patients after previous endovascular aortic repair. EuroSCORE levels were determined for all patients.

### The concept of distal shifting

Experience has taught that extensive manipulation of the left lung is a limiting factor during open thoracoabdominal replacement, and the more distally the repair can be begun, the better the operation is tolerated. Hence, in type I and type II TAAAs, aortic arch replacement using the frozen elephant trunk (FET) technique followed by thoracic endovascular aortic repair (TEVAR) or, in the case of a sufficient proximal landing zone, TEVAR alone as an initial step followed by metachronous open type III/type IV repair, is the treatment of choice.

### Anaesthesiological set-up and neuroprotection

Invasive blood pressure management is done at the right radial and at the right femoral arteries. Patients receive double-lumen intubation. A CSF drain is routinely inserted the day before surgery by an anaesthesiologist. Motor-evoked potentials (MEP) and somatosensory-evoked potentials (SSEP) are routinely monitored during the procedure. Near infrared spectroscopy is also applied on a routine basis.

### Surgical technique

Patients are placed in a right lateral supine position, and the operation is started with open surgical exposure of the femoral vessels. An 8 -mm Dacron graft is sewn to the common femoral artery to maintain distal perfusion during cardiopulmonary bypass (CPB). Afterwards, a thoraco-phrenico-lumbotomy is performed, and the viscera including the left kidney are placed to the right. Afterwards, depending on the extent of the repair, the distal aortic arch and the descending aorta are circumferentially dissected and encircled with silastic tapes. Then, CPB is established via the femoral vessels, the aorta is clamped at the most proximal level needed, haemodynamic equilibrium between the upper and lower body is achieved and sequential repair from proximal to distal is performed. Thoracic segmental arteries are routinely reimplanted. Which ones to preserve is decided before the operation based on the imaging results and during the operation under the guidance of the MEPs and SSEPs. When repairing the visceral and renal segments, normothermic blood perfusion through selective catheters via the cardioplegia pump is performed. We do not routinely measure flow to the respective end organs. Usually, the infrarenal anastomosis is done first so that we are able to re-establish circulation continuity between the upper and the lower body. The sequence of reimplantation usually is the right renal artery (RRA), the superior mesenteric artery (SMA), the coeliac trunk (CT) and the left renal artery. In type IV aneurysms, we do not use CPB or selective organ perfusion; we perform an oblique anastomosis to the RRA, SMA and coeliac trunk with direct reimplantation of the left renal artery into the main prosthesis.

### Surgical details in patients after thoracic endovascular aortic repair

During planning, if it is determined that there is a type IA or type III endoleak, large type II endoleaks are also considered. If there are no type IA or type III endoleaks, the strategy is to leave the stent graft in place, to clamp it (nitinol resumes its original shape after declamping) and to have an end-to-end anastomosis between a Dacron prosthesis and the stent graft. In large stent grafts, the collar of an inversed Siena prosthesis is used to correct the diameter. The side branches meant for the supraaortic vessels as well as the perfusion branch are used for the visceral and renal segments [13].

### Additional surgical procedures in infective situations/organ fistulations

In the case of native or prosthetic aortic infections, we follow a concept of “complete as possible” the removal of the indwelling prosthetic material, radical local debridement and continuity restoration with neoaortas made from bovine pericardium [14]. After aortic repair, the respective organ fistulation is addressed in the same surgical setting.

### Definition of unsuitability for endovascular repair

Unsuitability for TEVAR [11] was defined in terms of company instructions for use regarding endovascular fenestrated aortic repair and endovascular branched aortic repair as well as the individual experience of the aortic team taking all 4 dimensional aspects of the decision-making process into account.

### Statistical analyses

Continuous data are presented as the mean with the standard deviation in cases with a normal distribution; otherwise, they are presented as the median with quartiles (25th-75th). Normality distribution was tested with the Kolmogorov-Smirnov test. The *t*-test was applied to compare continuous variables in cases with an equal distribution; otherwise, the Mann-Whitney test was used. Categorical variables were compared using the χ^2^ test. In the case of a small group (*n* < 5), Fischer’s exact test was used. The Kaplan–Meier estimates and the log rank calculations were performed for survival and freedom from aortic-related reinterventions. Multivariate regression analysis was used to analyse the risk factors for in-hospital deaths. Statistical analyses were performed with GraphPad Prism V 8 (San Diego, CA, USA).

## RESULTS

### Patient demographics

Patient demographics are shown in Table 1. The mean patient age was 64.2 ± 12 years; 56 (70%) were men. The mean EuroSCORE was 7.9 ± 2.6 and was higher in the group with previous endovascular aortic repair (*P* = 0.021). The median body mass index was 25.6 (22.4 29.4). The diagnosis of Marfan syndrome was established in 9 patients (11.3%). Thirty-eight patients (47.5%) had an underlying diagnosis of aortic dissection. Of these, 22 (27.5%) had an aneurysm on the basis of a residual dissection after type A repair and 16 (20%) had an aneurysm due to a chronic type B aortic dissection. TAAA on the basis of a chronic type B aortic dissection occurred more often in the group with previous endovascular aortic repair (*P* = 0.095). The median aortic diameter was 70.5 (61.5; 88.3) cm in the group with previous endovascular aortic repair and 61 (57.0; 69.3) cm in the group with no previous endovascular aortic repair, respectively; the significant difference was *P* = 0.003. Two patients (2.5%) were on dialysis before the operation, as were all the patients in the group with previous endovascular aortic repair (*P* = 0.039).

**Table 1:** Patient demographics

Variables	Overall	Previous Endovascular aortic repair	No previous Endovascular aortic repair	*P*-value
	(n = 80)	(n = 26)	(n = 54)	
Age	64.2 ± 12	66.8 ± 9.1	65.5 ± 13.0	0.171
Sex (m)	56 (70%)	20 (76.9%)	36 (66.7%)	0.348
Diabetes	7 (8.8%)	2 (7.7%)	5 (9.3%)	1.000
Arterial hypertension	68 (85%)	22 (84.6%)	46 (85.2%)	1.000
Hyperlipidaemia	29 (36.3%)	7 (26.9%)	22 (40.7%)	0.229
EuroSCORE	7.9 ± 2.6	8.85 ± 2.7	7.4 ± 2.4	**0.021**
BMI	25.6, 22.4; 29.4	25.5, 23.1; 30.0	25.6, 22.3; 29.1	0.828
Marfan syndrome	9 (11.3%)	2 (7.7%)	7 (13%)	0.485
COPD	15 (18.8%)	7 (26.9%)	8 (14.8%)	0.194
Previous cerebrovascular events	11 (13.8%)	4 (15.4%)	7 (13 %)	0.742
Coronary artery disease	32 (40%)	14 (53.8%)	18 (33.3%)	0.079
Previous type A repair	22 (27.5%)	7 (26.9%)	15 (27.8%)	0.936
Type B aortic dissection	16 (20%)	8 (30.8%)	8 (14.8%)	0.095
Aortic diameter	63, 58.3; 75.0	70.5, 61.5; 88.3	61, 57.0; 69.3	**0.003**
Dialysis	2 (2.5%)	2 (7.7%)	0 (0.0%)	**0.039**
Urgent/emergency	19 (23.8%)	11 (42.3%)	8 (14.8%)	**0.007**
Elective	61 (76.2%)	15 (57.5%)	46 (85.2%)	**0.007**
Crawford I	16	7	9	0.372
Crawford II	17	3	14	0.242
Crawford III	26	9	17	0.803
Crawford IV	20	7	13	0.789
Crawford V	1	0	1	0.999

BMI: body mass index; COPD: chronic pulmonary disease.

Nineteen (23.8%) patients were operated on under urgent or emergency conditions, which occurred more often in the group with previous endovascular repair (42.3% vs 14.8%, *P* = 0.007). According to the modified Crawford classification, the extent of the thoracoabdominal aortic pathology was as follows: type I=16, type II=17, type III=26, type IV=20 and type V=1 with no intergroup difference.

### Previous aortic interventions/operations

Sixteen (20.0%) patients had previous FET implantation. Twenty-six patients (32.5%) underwent previous endovascular aortic interventions in various segments. Fifteen patients underwent TEVAR, 5 of them after a previous FET procedure; and 11 patients underwent a previous EVAR, 5 of whom also had TEVAR later (Table 2).

**Table 2:** Previous aortic interventions/operations

	Previous aortic interventions/operations, n (%)
FET	16 (20%)
Previous endovascular repair	26 (32.5%)
TEVAR	15 (18.6%)
TEVAR after FET	5 (6.3%)
EVAR	11 (13.8%)
TEVAR after EVAR	5 (6.3%)

EVAR: endovascular aortic repair; FET: frozen elephant trunk; TEVAR: thoracic endovascular aortic repair.

### Surgical details

Selective organ perfusion was performed in 63 patients (78.8%). The CPB time was 113.3 ± 80.1 min with no intergroup differences. Reimplantation of segmental arteries was performed in 40 patients (50%), again without intergroup differences. The need for blood transfusions and blood products was as follows: packed red blood cell units (PRBC) 10 (5; 14), fresh frozen plasma units 12 (7.5- 21.5) and platelet transfusion units, 4 (3–6). There was a significant difference in the need for PRBC between the groups, respectively (*P* = 0.014). The mean operating time was 406.4 ± 121.6 min. In 31 patients (38.8%), a straight Dacron prosthesis was used; in 25 patients (31.2%), a Coselli prosthesis (Terumo Aortic, Scotland, UK); and in 20 patients (25%), a Siena prosthesis (Terumo Aortic, Scotland, UK) was used. In 4 patients (5%) with native or prosthetic aortic infection, a self-made bovine pericardial graft used. All patients with xenopericardial grafts had previous endovascular aortic repair. The Siena prosthesis was more frequently used in the group with previous endovascular aortic repair. Five patients (6.3%) needed postoperative circulatory support with extracorporeal life support (Table 3).

**Table 3:** Intraoperative details comparing patients with previous endovascular aortic repair and no endovascular aortic repair

	Overall	Previous endovascular aortic repair	No previous endovascular aortic repair	*P*-value
	n = 80	n = 26	n = 54	
Selective organ perfusion using partial CPB	63 (78.8%)	24 (92.3%)	39 (72.2%)	**0.045**
Partial CPB bypass time (min)	113.3 ± 80.1	117.1 ± 71.65	108.8 ± 84.82	0.173
Segment artery reimplantation	40 (50%)	10 (38.5%)	30 (55.6%)	0.232
PRBC	10, 5; 14	11, 8; 20	8, 4; 12	**0.014**
FFP	12, 7.5; 21.5	12, 9; 21	13, 6.6; 23	0.797
PT	4, 3; 6	4, 2; 5	4.5, 3.8; 6.3	0.098
Operating time	406 ± 121.6	431.8 ± 149.6	407.0 ± 122.7	0.414
Straight Dacron prosthesis	31 (38.8%)	6 (23.1%)	25 (46.3%)	**0.046**
Coselli prosthesis	25 (31.2%)	3 (11.5%)	22 (40.7%)	**0.001**
Siena prosthesis	20 (25%)	13 (50%)	7 (13%)	**<0.001**
Self-made bovine pericardial graft	4 (5%)	4 (15.4%)	0 (0%)	**<0.001**
ECLS	5 (6.3%)	2 (7.7%)	3 (5.6%)	0.658

CPB: cardiopulmonary bypass; ECLS: extracorporeal life support; FFP: fresh frozen plasma units; PRBC: packed red blood cells, units; PT: platelet transfusion, units.

### Perioperative outcome

Overall, in-hospital mortality was 20% (*n* = 16). Permanent paraplegia was observed in 4 patients (5%). Stroke was seen in 5 patients (6.3%), and a tracheostomy was performed in 19 (23.8%) patients due to postoperative respiratory insufficiency. Intermittent haemodialysis for postoperative acute kidney injury was needed in 15 patients (18.8%) (Table 4). Eight patients died of multiorgan failure; 3 patients, of sequelae of haemorrhagic shock; 2, of septic shock; 2 died on the table during the operation for TAAA rupture; 1 died of severe intraoperative apoplexy.

**Table 4:** Postoperative outcome according to the Crawford type

Variables	Overall	Type I	Type II	Type III	Type IV	Type V
	n = 80	n = 16	n = 17	n = 26	n = 20	n = 1
In-hospital deaths	16 (20%)	4 (25%)	4 (23.5%)	6 (23.1%)	2 (10%)	0 (0%)
Paraplegia	4 (5%)	1 (6.3%)	1 (5.9%)	1 (3.8%)	1 (5%)	0 (0%)
Stroke	5 (6.3%)	1 (6.3%)	1 (5.9%)	3 (11.5%)	0 (0%)	0 (0%)
Tracheostomy	17 (21.3%)	5 (31.3%)	2 (11.8%)	7 (26.7%)	3 (15%)	0 (0%)
Intermittent need of haemodialysis	15 (18.8%)	3 (18.8%)	3 (17.7%)	6 (23.1%)	3 (15%)	0 (0%)

### Follow-up and need for aortic-related reinterventions

**Figure 1.**
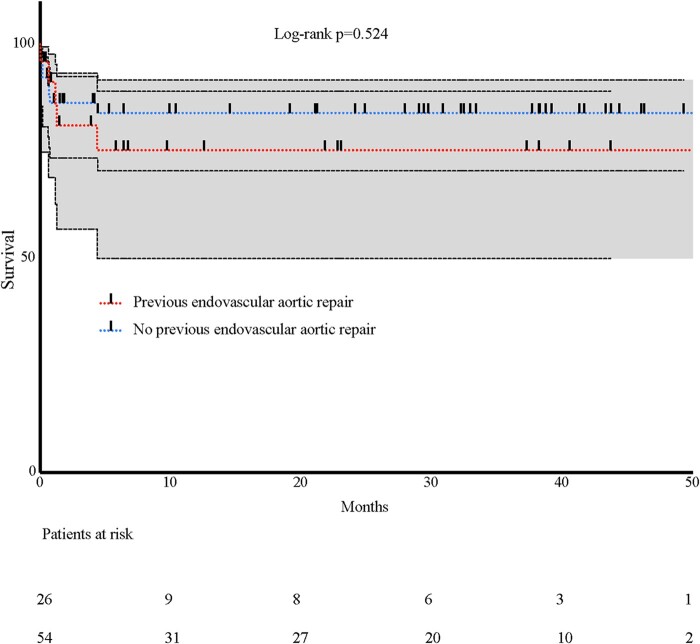
Kaplan–Meier survival curve comparing survival in patients with previous endovascular treatment and no previous endovascular treatment.

**Figure 2.**
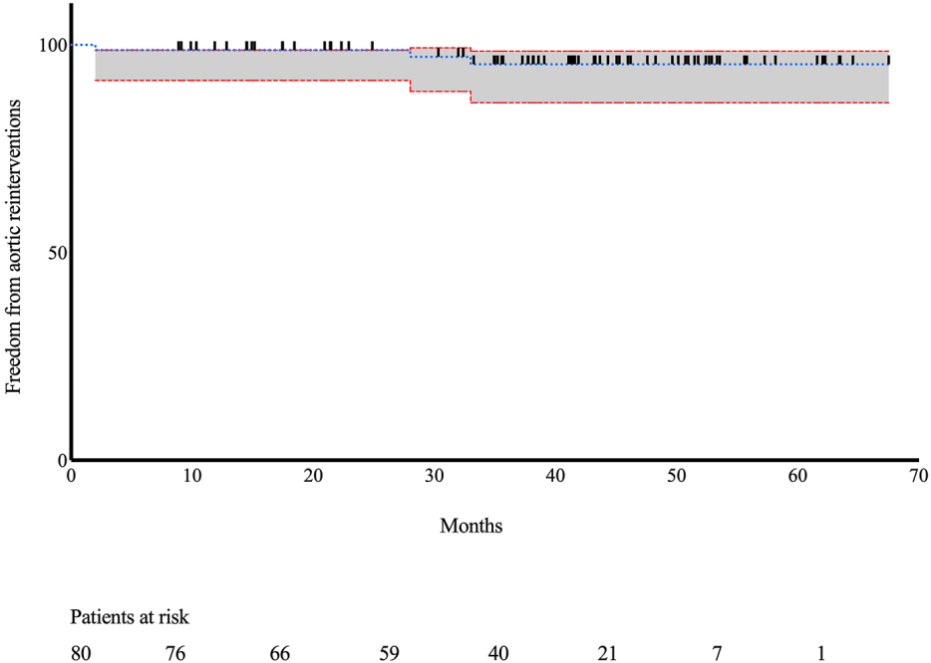
Freedom from aortic-related reinterventions.

Follow-up was 100%. Patients were followed in our aortic outpatient clinic by computed tomography angiography and clinical examinations. The mean follow-up was 37.9 ± 15.8 months. Survival was comparable in patients with previous endovascular aortic repair compared with that in patients with no previous endovascular aortic repair (log rank *P* = 0.524) (Fig. 1). Freedom from aortic-related reintervention during follow-up was 96.3% (Fig. 2). Three additional patients died of non-aortic related causes during the follow-up period: 1 patient died of a malignant tumour 16 months after surgery, and 2 died 18 months after surgery.

### Emergency/urgent versus elective procedures

When comparing emergency/urgent and elective procedures, more deaths occurred in the emergency/urgent group, and the need for tracheostomy was higher. However, paraplegia rates were comparable (Supplemental Table 1).

### Predictors of in-hospital deaths

Using multivariate logistic regression, the quantity of PRBC units (*P* = 0.009, CI 1.028–1.215, OR = 1.117) used was identified as a significant risk factor for in-hospital death (Table 5).

**Table 5:** Multivariate logistic regression for in-hospital deaths

	Odds ratio	CI	*P*-value
Sex	1.498	0.298-7.520	0.624
Age	1.011	0.942-1.085	0.767
COPD	0.278	0.076-2.098	0.278
Type A dissection	0.228	0.055-1.193	0.083
Type B dissection	2.147	0.309-14.911	0.440
Marfan syndrome	0.159	0.019-1.372	0.095
PRBC	1.117	1.028-1.215	**0.009**

COPD: chronic obstructive pulmonary disease; PRBC: packed red blood cells.

### Outcome according to previous endovascular aortic repair versus no previous endovascular aortic repair

We did not find any statistically significant differences when we compared outcomes between patients with previous endovascular aortic repair and no previous endovascular aortic repair. Table 6 shows perioperative mortality, morbidity and neurologic injury in the 2 groups.

**Table 6:** Outcome in the patient groups with previous endovascular treatment and no previous endovascular treatment

	Overall	Previous endovascular aortic repair	No previous endovascular aortic repair	*P*-value
	n = 80	n = 26	n = 54	
In-hospital deaths	16 (20%)	6 (23.1%)	10 (18.5%)	0.633
Paraplegia	4 (5%)	1 (3.9%)	3 (5.6%)	0.999
Stroke	5 (6.3%)	2 (7.7%)	3 (5.6%)	0.999
Tracheostomy	17 (21.3%)	6 (23.1%)	11 (20.4%)	0.988
Intermittent need of haemodialysis	15 (18.8%)	5 (19.2%)	10 (18.5%)	0.940

Data availability statement: All relevant data are within the manuscript and its supporting information files.

## DISCUSSION

Nearly 50% of patients had an underlying diagnosis of aortic dissection. Patients with aneurysmal formation on the basis of a residual dissection after a previous type A repair as well as patients with primary type B aortic dissections were equally distributed. This result mirrors the change in treatment strategies because many patients with degenerative aneurysmal formation now undergo primary endovascular fenestrated aortic repair or endovascular branched aortic repair, and few are indicated for primary surgery even if current recommendations would substantiate it [15–18]. One-fourth of the patients in this series were operated on under urgent and emergency conditions whereas there was a substantially higher number of patients with previous endovascular repair than without previous endovascular repair. This finding further underlines the need for continuing surveillance of patients after previous repair, in particular after endovascular repair, because failure is frequent, and obviously, in many settings, patients are not subjected to a stringent routine follow-up protocol [3, 18].

Previous aortic interventions and operations in this series occurred frequently at every level, whereas previous endovascular repair occurred most frequently, both TEVAR and endovascular aortic repair. The need for secondary open conversion is often attributed to trade-offs when primarily indicating treatment. The broad availability of endovascular therapy often supports choosing this treatment modality as the primary one. However, trade-offs regarding the adequate length of landing zones as well as respecting the anatomy in combination with the—by nature—higher probability of developing endoleaks by the higher number of modular components often lead to failure, early and late [7]. Finally, a broader application of the FET technique provides an ideal platform for secondary distal interventions, operations or both [3, 19]. The three-stage concept has recently been introduced as a safe, reproducible and reliable concept for treating mega-aortic syndromes, FET, TEVAR and open distal completion. Distal shifting of the disease by TEVAR extension reduces manipulation of the left lung during open thoracoabdominal replacement, which is one of the major components that influences the success or failure of this operation. Because many patients in our setting had a previous TEVAR and because many of them had been given large stent grafts, diameter correction between the stent graft and the Dacron graft used for distal extension is needed. The modification and inversed use of the Siena prosthesis has turned out to be an excellent means to accomplish this goal.

Perioperative mortality remains substantial but has to be interpreted in light of the surrounding conditions. Clearly, refraining from treating urgent and emergency cases would have enabled a lower perioperative mortality, but we are convinced that it is the responsibility of academic tertiary care centers to offer treatment to all comers even if the initial probability of treatment success is relative [21]. The paraplegia rate in this series was low and mirrors our concept of the routine use of CSF drainage, the preservation of major spinal cord inflow and the critical segmental arteries and the routine intraoperative monitoring of MEPs and SSEPs. Finally, we directly monitor spinal cord perfusion pressure and depict that on the haemodynamic monitor, which enables early intervention in case of borderline arterial pressure conditions [22].

The use of CPB varies in the literature depending on the TAAA type and on the need for sufficient organ protection. In our patient group, organ protection using CPB was performed in 63 patients (78.8%). The left heart bypass has advantages regarding intraoperative anticoagulation, amount of bleeding and other conceptual components. The major challenge is volume management, which is easier (and eventually safer) with partial CPB because volume shifts can be balanced better if pump suction and immediate reinfusion are available. Our concept was to stick with the partial CPB in types I, II, III and V. In type IV, we routinely clamp and sew an oblique anastomosis to the RRA, SMA and coeliac trunk with selective reimplantation of the left renal artery.

We identified the number of PRBC units as independent predictors of perioperative mortality. This approach mirrors the detrimental effect of high-volume turnover and the associated reduction of immunocompetence in the early postoperative period. Clearly, urgent or emergency scenarios are by nature associated with a high turnover, but in elective settings, every effort should be made to optimize blood management and to reduce the need for plasmatic and cellular transfusions to a minimum [1].

When comparing emergency/urgent and elective procedures, we observed higher mortality rates among emergent cases, which is not surprising. A positive surprise was that we did not observe a higher paraplegia rate in those having emergency/urgent procedures. We think that this finding is due to our stringent strategy of CSF drainage in all cases except in those in haemodynamic instability due to rupture. The stroke rate was higher in elective cases, and strokes occurred more frequently in patients with type I or type II extent compared to others. We developed a new strategy in these cases: We clamped the proximal aortic segment before initiation of CPB, which obviated retrograde dislodgement of atherosclerotic/thrombotic debris. This strategy has turned out to be successful. Tracheostomy rates were higher in emergency/urgent procedures. It is our strategy to go for an early tracheostomy because achieving respiratory weaning is more straightforward. Because we follow a distal shifting strategy (if feasible) by FET and secondary TEVAR extension, which leaves a 3.5 repair for the remaining segments, and because a no-touch technique for the left lung was followed, the incidence of severe respiratory failure needing prolonged weaning has substantially decreased.

Interestingly, we did not observe differences regarding outcome in patients with or without previous endovascular interventions. This outcome is a very positive aspect because many settings deem patients with previous extensive endovascular repair inoperable, which obviously is not the case [7]. However, strategies have to be developed to cope with the unique challenges associated with secondary surgical conversions after failed endovascular repair.

Follow-up in this series is complete due to a stringent surveillance in our outpatient clinic where all aortic patients with aortic diseases are actively followed. This approach is in particular instrumental in identifying the few patients who need secondary repair of any kind after thoracoabdominal replacement, which is fortunately an extremely rare occurrence. Finally, even in the modern endovascular era the open procedure remains a durable solution with a low rate of aortic reinterventions.

### Limitations

This report contains all the inherent limitations of a retrospective analysis. However, the unique advantage of this report is that it describes the open surgical treatment approach in an era where alternatives are available and are used, which naturally leaves a more complex patient cohort for open surgery. Finally, it is an all-comers series and does not preselect either by underlying pathology, frame conditions or urgency.

## CONCLUSION

Despite an early attrition rate, survival after open thoracoabdominal aortic replacement is excellent, and freedom from aortic-related reintervention is high. Open surgery continues to remain an essential component in the treatment armamentarium for acute and chronic thoracoabdominal aortic pathology.

## Funding

No outside funding was used for this project.

Conflict of interest: Martin Czerny and Bartosz Rylski are consultants to Terumo Aortic and shareholders of Ascense Medical, Martin Czerny is consultant to Medtronic, Endospan and NEOS, received speaking honoraria from Cryolife-Jotec and Bentley and is shareholder of TEVAR Ltd.

## Supplementary Material

ivac076_Supplementary_Data

## Abbreviations

CPB: cardiopulmonary bypass
FET: frozen elephant trunk
MEP: motor-evoked potentials
PRBC: packed red blood cells units
RRA: right renal artery
SMA: superior mesenteric artery
SSEP: somatosensory-evoked potentials
TAAA: thoracoabdominal aortic aneurysm
TEVAR: thoracic endovascular aortic repair
