# Supplementary material for: Results of open thoracoabdominal aortic replacement in patients unsuitable for or after endovascular repair with remaining disease components
Source: Interact Cardiovasc Thorac Surg. 2022 Apr 19;35(3):ivac076. doi: 10.1093/icvts/ivac076 (PMC9419677; doi:10.1093/icvts/ivac076)
Supplement: ivac076_Supplementary_Data [file ivac076_supplementary_data.docx]

Supplemental Table 1. Outcome in the patient groups with elective vs emergent or urgent open TAAA repair.

|  | Overall  n=80 | Emergent or urgent  n=19 | Elective  n=61 | P-value |
| --- | --- | --- | --- | --- |
| In-hospital mortality | 16 (20%) | 8 (31.6%) | 10 (16.4 %) | **0.019** |
| Paraplegia | 4 (5%) | 1 (5.2%) | 3 (4.9%) | 0.999 |
| Stroke | 5 (6.3%) | 0 (0 %) | 5 (8.1%) | 0.331 |
| Tracheostomy | 17 (21.3%) | 8 (42.1%) | 9 (14.8%) | **0.011** |
| Intermittent need of hemodialysis | 15 (18.8%) | 1 (5.3%) | 14 (22.9%) | 0.103 |
|  |  |  |  |  |
